# The impact of COVID-19 quarantine on lifestyle indicators in the United Arab Emirates

**DOI:** 10.3389/fpubh.2023.1123894

**Published:** 2023-02-13

**Authors:** Sharifa AlBlooshi, Maryam AlFalasi, Zainab Taha, Farid El Ktaibi, Alia Khalid

**Affiliations:** ^1^Department of Health Sciences, College of Natural and Health Sciences, Zayed University, Dubai, United Arab Emirates; ^2^Department of Health Sciences, College of Natural and Health Sciences, Zayed University, Abu Dhabi, United Arab Emirates; ^3^Department of Mathematics and Statistics, College of Natural and Health Sciences, Zayed University, Abu Dhabi, United Arab Emirates

**Keywords:** COVID-19, physical activity, weight, dietary habits, food consumption, smoking, sleeping patterns, food purchasing patterns

## Abstract

**Introduction:**

COVID-19 is a virus that has spread rapidly and brought economic and social crises all around the world. The current study aimed to investigate the impact of COVID-19 quarantine on dietary habits, physical activity, food purchasing, smoking, and sleeping patterns in the United Arab Emirates.

**Methods:**

A cross-sectional study was conducted using an online questionnaire between November 1st, 2020 and the end of January 2021. Citizens and residents of the UAE aged ≥ 18 years old were asked to complete an anonymous electronic questionnaire created via Google Forms and distributed on various platforms, such as WhatsApp, Twitter, and email. A total of 1682 subjects participated in the study.

**Results:**

The results included that during the COVID-19 lockdown, more participants (44.4%) reported an increase in weight. This gain seems to be linked to increased food consumption [(Adjusted Odd Ratio) AOR = 1.68, 95% (Confidence Interval) CI = 1.12, 2.54, *p* = 0.022], decreased physical activity (AOR = 2.25, 95% CI = 1.58, 3.21, *p* < 0.001), and increased smoking (AOR = 1.90, 95% CI = 1.04, 3.50, *p* = 0.038). The groups that were most likely to gain weight included those who consumed more cereals (AOR = 1.67, 95% CI = 1.08, 2.57, *p* = 0.011), had an increased desire for sweets (AOR = 2.19, 95% CI = 1.50, 3.19, *p* < 0.001), and an increased desire for food (hunger) (AOR = 2.19, 95% CI = 1.53, 3.14, *p* < 0.001). In contrast, those who exercised more were more likely to lose weight (AOR = 0.61, 95% CI = 0.44, 0.86, *p* < 0.001) as well as those who slept over 9 h a day (AOR = 1.90, 95% CI = 0.45, 0.88, *p* = 0.006).

**Discussion:**

Overall, it is essential to promote healthy habits and methods of healthy diet maintenance during stressful and unusual times when people might find it difficult to put effort into their health.

## 1. Introduction

COVID-19 is an infectious disease caused by a newly discovered strain of coronavirus; a type of virus known to cause respiratory infections in humans. This new strain was unknown before December 2019, when an outbreak of pneumonia of unknown cause emerged in Wuhan, China (1). On March 11th, 2020, the World Health Organization (WHO) (1) declared that COVID-19 is a worldwide pandemic as the disease had spread enormously worldwide.

To combat COVID-19, many countries have applied preventive measures such as disinfection procedures and partial or complete lockdowns to slow the spread of the virus. For example, the United Arab Emirates (UAE) health authorities implemented preventative measures to protect public health in line with the WHO rules and regulations. On March 1st, 2020, the UAE authorities applied strict infection control and partial lockdown for more than 6 months, forcing many people to stay home (study and work from home). Nevertheless, such actions may cause a sudden and drastic change in the population's lifestyle. It has been reported that staying at home for a long time may lead to a change in dietary habits, physical activity, and sleep patterns, as well as psychological impact (2).

The economic impact of COVID-19 on diet quality and food security is also a concern. Healthy and nutrient-rich foods have become increasingly unaffordable and inaccessible, especially to people of lower socioeconomic status and informal workers (3).

The alteration of some life aspects and the new routine of people's daily life makes researchers want to study people during this critical period. For example, staying at home while news spreads about the COVID-19 pandemic can generate unpleasant emotions such as boredom, anxiety, and stress. These emotions are linked to increased food intake, especially “comfort” food known to be high in sugar and fat (4).

Such patterns have already been observed in several populations. Studies found that following the lockdown, eating habits and physical activity were negatively impacted (5–15). People reported an increase in main meals, frequent snacking, and experiencing a lack of motivation and control regarding food (5, 6). Additionally, they also showed reduced levels of physical activity and increased sedentary behavior. This suggests that many individuals cannot maintain appropriate levels of physical activity during quarantine (6–9). Likewise, studies done in the UAE found similar results (11–13). It is essential to determine the extent of such patterns in the UAE to address them and prevent the deterioration of the population's health.

As previously stated, the emergence of the COVID-19 pandemic has impacted almost every facet of life, including people's access to food and goods (16). As a result, many individuals have limited their outings, opting to shop for food online (17). During the height of the pandemic, many restaurants around the world were shuttered. However, delivery services were still available, which led to an increase in the usage of food delivery applications. Many individuals chose to have their groceries delivered to their homes to avoid crowds (18–22). This was especially true for those of higher income, higher levels of education, and those who find food-related online channels easier to use (16, 23). Similar patterns have been examined in the Middle East, including the UAE (24–31).

However, some populations appear to have some concerns regarding online food shopping. A study done in Brazil found that the preparation method was the primary worry among those who wouldn't rely on food delivery (32). Other studies also found that many responders were concerned about being unable to check the freshness and quality of the product when purchasing groceries online (26, 33). In addition, it was determined from a study conducted in Portland, United States, that lower-income consumers are less likely to employ internet delivery services (16). Furthermore, consumers stated that the complexity of using online tools for purchasing food goods alongside technological issues decreases their proclivity to use or re-use these technologies (23).

Another health aspect that has been influenced by the COVID-19 lockdown is smoking. The self-isolation induced by the COVID-19 pandemic seems to have increased the consumption of cigarettes per day (34). A study conducted in the UAE reported a rise in smoking in 21% of its 2060 respondents (13). Similar results have been determined in other studies around the world. This increase may be tied to the heightened stress levels during the COVID-19 pandemic (13, 35–38). However, findings included conflicting results where large percentages of participants did not change their smoking habits, reduce them, or even quit smoking entirely (35–38). A study in Italy attributed the reduction in smoking among its participants to their fear of the COVID-19 mortality risk (35).

Additionally, studies on the COVID-19 pandemic show that many people have been experiencing sleep difficulties that did not exist before the pandemic (39–43). The studies' populations commonly show a reduction in night-time sleeping, an increase in day-time napping, and a shift to a later bedtime (39, 42, 43). In addition, some people have slept more hours overall, but the quality of their sleep has declined (34, 37). For example, in a study done in the UAE, decreased sleep was reported among 20.8% of the 2,060 responders (13). Research also indicates that younger people and women were most likely to report sleep distress that may have arisen due to psychological distress during the COVID-19 pandemic (13, 43–45).

The novel coronavirus (COVID-19) pandemic has brought extraordinary challenges in various aspects of life. As a result, the United Arab Emirates has imposed stringent rules, including a lockdown that extended a nationwide daily curfew. Since the UAE is a multinational country, our results would be of great importance to health authorities when revising their health policies in pointing out the consequences on the local Emirati people. Therefore, it is important to investigate the consequences of the COVID-19 pandemic and quarantine on the health of the UAE population to create targeted interventions to improve people's lifestyles following the pandemic and to prevent similar outcomes in the case of emergencies. This is especially needed in the UAE because reports of unhealthy lifestyles were already high before the COVID-19 pandemic (13). This study aims to investigate the impact of COVID-19 quarantine on several health-related aspects among adults in the UAE. These aspects are dietary habits, physical activity, food purchasing patterns, smoking, and sleeping patterns.

## 2. Materials and methods

### 2.1. Study design

This study used a quantitative cross-sectional study design with a questionnaire as a data collection tool.

### 2.2. Sampling and recruitment

This study used snowball sampling. The inclusion criteria were citizens and residents of the UAE aged ≥18 years old, both male and female. Participants were asked to complete an anonymous electronic questionnaire created *via* Google Forms and distributed on various platforms, such as WhatsApp, Twitter, and email. The questionnaire link was sent in Arabic and English for the participants to use the language they prefer as some preferred English language, this has been concluded from the pilot study. The principal investigator sent the questionnaire *via* email to students and to other Zayed University (ZU) faculties. It was shared with students and faculties in different colleges at ZU. Moreover, faculties sent it to their students *via* email and WhatsApp groups, and they were asked to share it with their friends and family members. We used Zayed University email addresses to reach the participants. The questionnaire was available between November 1st 2020 and the end of January 2021. A total of 118 questionnaires with missing answers were removed, and we ended with 1,682 completed questionnaires.

### 2.3. Data collection tool

An online questionnaire was designed to assess and explore changes in dietary habits, physical activity, food purchasing, smoking, and sleeping patterns during the COVID-19 pandemic in the UAE. This questionnaire was adopted from another similar study that used the questionnaire as a measurement tool. It has been modified from a survey by Di Renzo, which investigated the impact of the COVID-19 pandemic on eating habits and lifestyle changes among the Italian population aged ≥12 years. The study comprised a structured questionnaire that inquired about demographic information, anthropometric data (reported weight and height); dietary habits information and lifestyle habits information (2). The survey comprised 3 main sections with 32 questions in total. The platform used was Google Forms, and the link to the questionnaire was shared *via* WhatsApp, Twitter, Snapchat, and Instagram. The questionnaire was initially developed in English, then translated into Arabic and then pilot-tested with 30 students that were not familiar with the subject (not from the College of Natural and Health Sciences), and the errors were reviewed by the authors.

The first section included 10 questions regarding demographic data such as age, gender, nationality, occupation, medical history, weight, and height. The second section had 16 questions regarding dietary habits, such as the type of food consumed, the number of meals, and snacks. This section focused on assessing the participants' dietary intake during the lockdown and whether it underwent any changes. The third section had six questions regarding lifestyle habits such as exercise, smoking, and purchasing daily necessities. Individuals were also asked if their physical activities and weight had changed after the lockdown (after August 2020), COVID-19 lockdown period was defined as March 1st, 2020 to the End of August 2020 as per the Supreme Council for the National Security Emergency Crises and Disasters Management Authority in the UAE in August 2020.

### 2.4. Data analysis

Data were analyzed using the Statistical Package for Social Sciences, SPSS Version 27. Frequency distributions and percentages generated descriptive statistics to analyze the general characteristics of the participants. To better understand the relationship (association) between the dependent variable (weight change) and the independent variables (number of daily meals, number of snacks per day, consumption of cereals, consumption of sources of protein, consumption of fruits and vegetables, consumption of sweets and French fries, sense of hunger and satiety, level of physical activity), multiple Chi-square tests or Fisher exact tests were conducted whenever appropriate. Additionally, a contingency table was constructed to detail the food intake of the participants.

### 2.5. Ethical clearance

This study was approved by the Research Ethics Committee at Zayed University, UAE (ZU20_137_F) and the Research Ethics Committee at the Ministry of Health and Prevention (MOHAP/DXB-REC/ONN/No.147/2020). All study participants provided informed consent at the beginning of the online questionnaire.

## 3. Results

### 3.1. Characteristics of the study participants

A total of 1,682 participants were included in the analysis. Table 1 presents the general characteristics of the studied population. Most participants were aged 18–29 years old (69.7%), female (80.9%), from Dubai (51.4%), and the Northern Emirates (Sharjah, Ajman, Fujairah, Ras al-Khaimah, and Umm al-Quwain) (37.8%). Approximately half of the participants had a high school education (51.6%), and the rest had a university-level and higher education (45.4%). Around half of the participants were students (46.4), 19.1% were unemployed, and the rest were employed. In addition, the majority had a monthly income of <5,000 AED (63.2%).

**Table 1:** General characteristics of the study participants (*n* = 1,682).

**Variables**	***N***	**%**
**Age (years)**		
18–29	1,172	69.7
30–39	253	15.1
40–49	190	11.3
50–59	63	3.7
≥ 60	4	0.2
**Sex**		
Male	322	19.1
Female	1,360	80.9
**Emirate**		
Abu Dhabi	181	10.8
Dubai	866	51.4
Sharjah	271	16.1
Ajman	200	11.9
Umm Al Quwain	104	6.2
Ras Al Khaimah	45	2.7
Fujairah	15	0.9
**Employment status**		
Student	780	46.4
Employed full-time	468	27.8
Employed part-time	44	2.6
Self-employed	38	2.3
Unemployed	322	19.1
Retired	30	1.8
**Education level**		
Below high school	50	3
High school	868	51.6
University education	662	39.3
Higher education	102	6.1
**Income (AED** ^*^ **)**		
< 2,000	837	49.8
2,000–5,000	226	13.4
5,000–10,000	99	5.9
10,000–20,000	230	13.7
20,000–40,000	226	13.4
More than 40,000	64	3.8

* United Arab Emirates Dirham.

### 3.2. Changes in body weight during the COVID-19 lockdown

**Figure 1:**
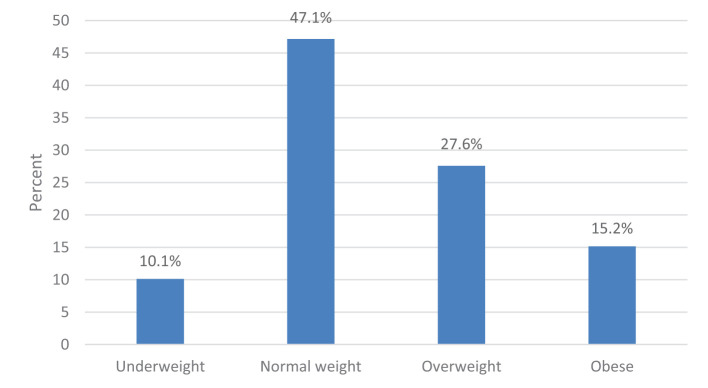
Participants' BMI during the COVID-19 lockdown (*n* = 1,682).

**Figure 2:**
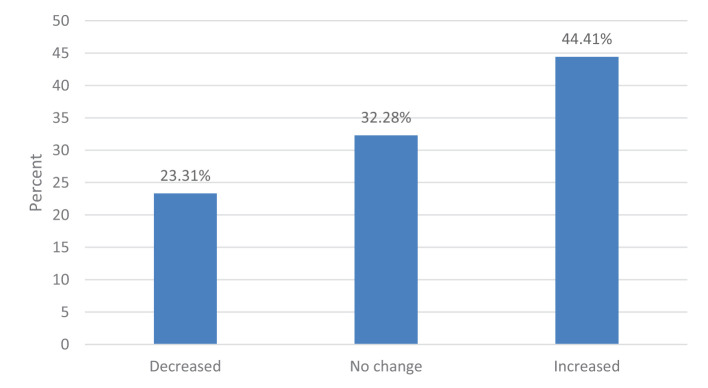
Participants' weight changes during the COVID-19 lockdown (*n* = 1,682).

Figures 1, 2 present the participants' BMI and weight changes, respectively, during the COVID-19 lockdown period which was defined as the 1st of March 2020 to the End of August 2020 as per the Supreme Council for the National Security, National Emergency Crises and Disasters Management Authority in the UAE in August 2020. According to BMI categories, the majority of the participants had a normal weight (47.1%), followed by overweight (27.6%), obese (15.2%), and underweight (10.1%). Regarding weight changes during the COVID-19 lockdown, most participants (44.4%) reported an increase in weight, while 23.3% reported weight loss and 32.3% reported no change.

In Table 2, the changes in weight during the COVID-19 lockdown according to age, gender, level of education, employment status, and monthly income are illustrated. Firstly, there was a significant association between changes in weight during the COVID-19 lockdown and age (*p* = 0.002). The youngest age group, aged 18–29 had the highest frequency across all weight change categories (gained, lost, and no change), with the highest being for weight loss (76.8%) followed by weight gain (69.3%). Those aged 30–39 had the second highest frequency for weight gain (15.8%), which was also the highest frequency for this age group across the weight change categories. Individuals over 60 made up the lowest frequency across the weight change groups, with the highest being for weight loss (0.5%).

**Table 2:** Changes in weight during COVID-19 according to age, gender, level of education, employment status, and monthly income (*n* = 1,682).

	**Total** **(*n* = 1,682)**		**Weight change**		***p*-value**
		**Weight loss** **(*****n*** = **392)**	**No change** **(*****n*** = **543)**	**Weight gain** **(*****n*** = **747)**	
Age					0.002
18–29	1,172 (69.7)	301 (76.8)	353 (65.0)	518 (69.3)	
30–39	253 (15.0)	51 (13.0)	84 (15.5)	118 (15.8)	
40–49	190 (11.3)	24 (6.1)	78 (14.4)	88 (11.8)	
50–59	63 (3.7)	14 (3.6)	27 (5.0)	22 (2.9)	
Over 60	4 (0.2)	2 (0.5)	1 (0.2)	1 (0.1)	
Gender					0.009
Male	322 (19.1)	55 (14.0)	119 (21.9)	148 (19.8)	
Female	1,360 (80.9)	337 (86.0)	424 (78.1)	599 (80.2)	
Highest level of education					0.021
Below high school	50 (3.0)	10 (2.6)	20 (3.7)	20 (2.7)	
High school	868 (51.6)	207 (52.8)	258 (47.5)	868 (53.9)	
Bachelor's degree	662 (39.4)	161 (41.1)	233 (42.9)	662 (35.9)	
Graduate level	102 (6.1)	14 (3.6)	32 (5.9)	102 (7.5)	
Employment status					< 0.001
Student	780 (46.4)	225 (57.4)	228 (42.0)	327 (43.8)	
Employed full-time	468 (27.8)	70 (20.7)	96 (29.8)	225 (30.1)	
Employed part-time	44 (2.6)	90 (3.1)	157 (1.8)	22 (2.9)	
Self-employed	38 (2.3)	93 (2.0)	173 (2.8)	15 (2.0)	
Unemployed	322 (19.1)	60 (15.3)	117 (21.5)	145 (19.4)	
Retired	30 (1.8)	6 (1.5)	11 (2.0)	13 (1.7)	
Monthly income (AED^*^)					0.026
< 2,000	837 (49.8)	226 (57.4)	260 (47.9)	351 (47.0)	
2,000–5,000	226 (13.4)	51 (13.0)	74 (13.6)	101 (13.5)	
5,000–10,000	99 (5.9)	22 (5.6)	30 (5.5)	47 (6.3)	
10,000–20,000	230 (13.7)	32 (8.2)	80 (14.7)	118 (15.8)	
20,000–40,000	226 (13.4)	45 (11.5)	77 (14.2)	104 (13.9)	
More than 40,000	64 (3.8)	6 (4.1)	22 (4.1)	26 (3.5)	

* United Arab Emirates Dirham.

Regarding gender, there was a significant association between changes in weight during the COVID-19 lockdown and gender (*p* = 0.009). Females had the highest frequencies in all weight change categories, with weight loss being the highest (86.0%) followed by weight gain (80.2%). Males made up a bigger percentage of the “no change” category (21.9%) compared to their frequency in weight gain (19.8%) and weight loss (14.0%). Level of education was also significantly associated with changes in weight during the COVID-19 lockdown (*p* = 0.021). The majority possessed a high school degree (51.6%). This group was the most frequent in all weight change categories, with the highest being in weight gain (53.9%) closely followed by weight loss (52.8%). Those with a bachelor's degree had their highest frequency in the no-change group (42.9%). Those below high school also had their highest frequency in the no-change group (3.7%). Finally, those at the graduate level had their highest frequency in the weight gain group (7.5%).

There was a significant association between weight changes during the COVID-19 lockdown and employment status (*p* < 0.001). Those who gained weight mainly were students (43.8%), followed by employed full-time (30.1%). The lowest percentage was retired people (1.7%). Among those who lost weight, most were students (57.4%), followed by employed full-time (20.7%). The last were retired (1.5%). Finally, those who experienced no change were mainly students (42.0%), then employed full-time (29.8%). The smallest percentage was retired (2.0%). Finally, monthly income is another variable significantly associated with weight changes during the lockdown (*p* = 0.026). Those who earned <2,000 AED had the highest frequency in all weight change categories. Among those, the highest was for weight loss (57.4%). The second highest frequency for weight gain, after those who earned <2,000 AED (45.0%), was for those who earned 10,000–20,000 AED (15.8%). This percentage was also the highest for this group across all weight change categories, meaning that this group mostly gained weight.

Table 3 illustrates the relationship between changes in dietary habits (such as the frequency of meals, snacks, and the consumption of certain food groups) and the weight change groups (gain, loss, or no change). Approximately 35.7% of those who gained weight during quarantine showed an increase in the number of daily meals. Compared to 14.5% of those who lost weight and 14.5% of those who experienced no change. Similarly, the highest frequency of increased snacking 304 (40.7%) was in individuals who gained weight. Changes in the number of daily meals were significantly associated with changes in weight during the COVID-19 lockdown (*p* < 0.001).

**Table 3:** Changes in dietary habits compared with weight changes during the COVID-19 lockdown in UAE (*n* = 1,682).

	**Total** **(*n* = 1,682)**		**Weight change**		***p*-value**
		**Weight loss** **(*****n*** = **392)**	**No change** **(*****n*** = **543)**	**Weight gain** **(*****n*** = **747)**	
The number of daily meals					< 0.001
Decreased	512 (30.4)	176 (44.9)	133 (24.5)	203 (27.2)	
No change	767 (45.6)	159 (40.6)	331 (61.0)	277 (37.1)	
Increased	403 (24.0)	57 (14.5)	79 (14.5)	267 (35.7)	
The number of snacks per day					< 0.001
Decreased	324 (19.3)	145 (37.0)	75 (13.8)	104 (13.9)	
No change	691 (41.0)	137 (34.9)	295 (54.3)	259 (34.7)	
Increased	471 (28.0)	66 (16.8)	101 (18.6)	304 (40.7)	
The consumption of cereals per day					< 0.001
Decreased	300 (17.8)	138 (35.2)	77 (14.2)	85 (11.4)	
No change	939 (55.8)	181 (46.2)	363 (66.9)	395 (52.9)	
Increased	331 (19.7)	39 (9.9)	58 (10.7)	234 (31.3)	
The consumption of sources of protein per day					< 0.001
Decreased	241 (14.3)	88 (22.4)	66 (12.2)	87 (11.6)	
No change	1,053 (62.6)	225 (57.4)	376 (69.2)	452 (60.5)	
Increased	289 (17.2)	59 (15.1)	63 (11.6)	167 (22.4)	
The consumption of fruits and vegetables per day					< 0.001
Decreased	280 (16.6)	78 (19.9)	63 (11.6)	139 (18.6)	
No change	829 (49.3)	166 (42.3)	318 (58.6)	345 (46.2)	
Increased	434 (25.8)	121 (30.9)	120 (22.1)	193 (25.8)	
The consumption of sweets and French fries per day					< 0.001
Decreased	334 (19.9)	139 (35.5)	92 (16.9)	103 (13.8)	
No change	672 (40.0)	137 (34.9)	269 (49.5)	266 (35.6)	
Increased	464 (27.6)	57 (14.5)	87 (16.0)	320 (42.8)	
The sense of hunger and satiety					< 0.001
Decreased appetite	392 (23.3)	207 (52.8)	89 (16.4)	96 (12.9)	
No change	508 (30.2)	98 (25.0)	280 (51.6)	130 (17.4)	
Increased appetite	782 (46.5)	87 (22.2)	174 (32.0)	521 (69.7)	

Consuming cereals, protein sources, fruits, and vegetables, sweets, and French fries during quarantine was significantly associated with changes in weight (*p* < 0.001 for all). Those who consumed more cereals and protein sources had their highest frequencies in the weight gain group (31.3% and 22.4%, respectively). On the other hand, those who increased their consumption of fruits and vegetables had the highest frequency in the weight loss group (30.9%). Most participants reported no change in their consumption of sweets and French fries (40.0%), while 27.6% reported an increase in consumption. However, 42.8% of those who gained weight reported an increase in consumption of this category.

During the quarantine, most participants reported an increase in appetite (46.5%). More than two-thirds (69.7%) of those who gained weight reported an increase in appetite. In contrast, 52.8% of those who lost weight reported a decrease in appetite. Changes in the sense of hunger and satiety were significantly related to changes in weight during the COVID-19 lockdown (*p* < 0.001).

Table 4 summarizes the changes in physical activity and smoking level compared to weight changes during the lockdown. Among the 1,682 participants, only 21.8% reported increased physical activity, 26.1% decreased physical activity, 22.8% reported no changes, and 29.4% never practiced physical activity. Among those who increased their physical activity (21.8%) during the lockdown, 38% lost weight, and 32% experienced no changes in their weight. On the other hand, among those who decreased their physical activity, 62.2% gained weight, and only 15.9% reported no body weight changes. Moreover, 41% reported no changes in their weight among those who didn't change their physical activity. Weight loss was reported by 23.5% of this group. There was a significant relationship between changes in levels of physical activity and changes in weight before and after lockdown (*p* < 0.001).

**Table 4:** Changes in the level of physical activity and smoking habits compared with weight changes during the COVID-19 lockdown in UAE (*n* = 1,682).

	**Total** **(*n* = 1,682)**		**Weight change**		***p*-value**
		**Weight loss** **(*****n*** = **392)**	**No change** **(*****n*** = **543)**	**Weight gain** **(*****n***= **747)**	
Level of physical activity					< 0.001
Decreased	439 (26.1)	70 (15.9)	96 (21.9)	273 (62.2)	
No change	383 (22.8)	90 (23.5)	157 (41.0)	136 (35.5)	
Increased	366 (21.8)	139 (38.0)	117 (32.0)	110 (30.1)	
Smoking change					0.033
Decreased	44 (2.6)	9 (2.3)	12 (2.2)	23 (3.1)	
No change	1,580 (93.9)	374 (95.4)	519 (95.6)	687 (92)	
Increased	58 (3.4)	9 (2.3)	101 (2.2)	304 (5.0)	

Similarly, changes in smoking habits were significantly associated with changes in weight during lockdown (*p* = 0.033). The majority reported no change in their smoking habits (93.9%). Those who increased their smoking had a higher frequency of weight gain (5.0%) than those who decreased their smoking (3.1%).

Table 5 presents the adjusted factors significantly associated with the change in weight. After adjusting for the other confounders, people who increased the number of meals consumed were more likely to gain weight (AOR = 1.68, 95% CI = 1.12, 2.54). While an increase in the cereals consumed was positively associated with the change in weight (AOR = 1.67, 95% CI = 1.08, 2.57), the persons who reduced the number of consumed cereals were more likely to lose weight (AOR = 0.53, 95% CI = 0.35, 0.81). The respondents who reported an increase in their sweets' consumption or their desire for food (hunger) had twice the odds of putting on more weight (AOR = 2.19, 95% CI = 1.50, 3.19) and (AOR = 2.19, 95% CI = 1.53, 3.14), respectively. On the other hand, compared to those who did not face any change in the desire for sweets or food (hunger), the persons who reported a decline in their sweets' consumption or their desire for food (hunger) are more likely to lose weight (AOR = 0.84, 95% CI = 0.58, 1.21) and (AOR = 0.54, 95% CI = 0.36, 0.81), respectively.

**Table 5:** Adjusted factors significantly associated with a change in weight.

**Change in weight**	**AOR**	**95% CI**		***p*-value**
**Meals consumed**				
No change				
Decreased	1.08	0.84	1.38	0.566
Increased	1.42	1.05	1.91	0.022
**Snacks**				
No change				
Decreased	0.72	0.54	0.97	0.031
Increased	1.04	0.78	1.39	0.774
**Cereals**				
No change				
Decreased	0.63	0.47	0.85	0.002
Increased	1.50	1.10	2.06	0.011
**Sweets and French fries**				
No change				
Decreased	0.81	0.61	1.07	0.142
Increased	2.21	1.68	2.90	< 0.001
**Sense of hunger and satiety**				
No change				
Decreased	0.50	0.37	0.66	< 0.001
Increased	2.83	2.18	3.68	< 0.001
**Physical activity**				
No change				
Decreased	1.66	1.29	2.14	< 0.001
Increased	0.50	0.39	0.65	< 0.001
**Sleeping hours**				
< 7 h per night				
7–9 h per night	0.88	0.70	1.12	0.295
More than 9 h per night	1.90	0.45	0.88	0.006
**Smoking change**				
No change				
Decreased	1.56	0.83	2.94	0.166
Increased	1.90	1.04	3.50	0.038

Physical activity was negatively associated with the change in weight: increasing the level of physical activity was more likely to lead to a loss in weight (AOR = 0.61, 95% CI = 0.44, 0.86). On the contrary, persons who practiced fewer sports activities had twice the odds of gaining weight (AOR = 2.25, 95% CI = 1.58, 3.21). The consumption of protein was not significantly associated with the change in weight.

When it comes to sleeping patterns, those who slept more than 9 h per night were more likely to lose weight (AOR = 1.90, 95% CI = 0.45, 0.88). In terms of changes in smoking, those who stated rising in smoking were more likely to gain weight (AOR = 1.90, 95% CI = 1.04, 3.50).

## 4. Discussion

The present study aimed to investigate the impact of COVID-19 quarantine on dietary habits, physical activity, food purchasing, smoking, and sleeping patterns in the UAE. Overall, this study has found that quarantine has negatively affected these health-related variables.

In terms of weight changes during the COVID-19 lockdown, most participants (44.4%) reported an increase in weight. This weight gain can be attributed to the general decrease in energy expenditure since quarantine limits people's ability to go to work, gyms, parks, and even to practice their regular daily routines. In addition, the emotional distress accompanied by having to remain at home for months, fear of novelty, and the high spread of COVID-19 might have provoked emotional eating and cravings (46). Other determinants leading to increased weight gain during the lockdown include prior behaviors, dietary habits, physical activity, type of work environment, psychosocial and socioeconomic factors, and co-morbidities (4). This result agrees with previous studies that evaluated weight gain relating to COVID-19 home confinement (2, 47, 48). In addition, studies in other countries revealed an increase in caloric intake and indicated weight gain during the COVID-19 lockdown (2, 49–51).

However, in this study, 32.3% of participants did not notice any weight change, and 23.3% reported weight loss. This could be due to high levels of awareness, or they may not have been as majorly affected by quarantine.

During the lockdown, those who gained weight had the highest frequency of participants who increased the number of their daily meals (35.7%), and the highest frequency of increased snacking (40.7%). This is compared to those who reported losing or no weight change. Further testing using logistic regression also showed that those who increased the number of consumed meals were more likely to gain weight (AOR = 1.68, 95% CI = 1.12, 2.54). Similarly, previous studies reported higher amounts of food intake during lockdown periods in Poland, Italy, and UK (5, 19). Consuming more cereals during quarantine was significantly associated with increased weight gain (*p* < 0.001) and with changes in weight (AOR = 1.67, 95% CI = 1.08, 2.57). Conversely, those who reduced the amount of consumed cereals were more likely to lose weight (AOR = 0.53, 95% CI = 0.35, 0.81). A significant difference was seen between the frequency of sweets and French fries' consumption and weight changes during quarantine (*p* < 0.001). The participants who reported increased intake of sweets had double the odds of putting on more weight (AOR = 2.19, 95% CI = 1.50, 3.19).

Furthermore, those who experienced a decline in their desire for sweets were more likely to lose weight (AOR = 0.84, 95% CI = 0.58, 1.21) than those who didn't experience a change. This was in line with a previous study in Germany which demonstrated an increase in the consumption of foods that are high in sugar and fat such as sweets, and found that it was a significant determinant of weight changes during quarantine (52). This may be due to the increased stress caused by the pandemic. People tend to seek “comfort foods” while coping with stressful situations (52). Furthermore, during home confinement, people tended to stock their kitchens with food to reduce unnecessary grocery trips out of fear of contracting the infection (48). The availability of large quantities of food for many days might lead to overeating that is not necessarily due to hunger (53).

During the lockdown, changes in hunger and satiety were significantly related to weight changes (*p* < 0.001). The current study showed that 69.7% of those who gained weight reported an increase in appetite. In contrast, 52.8% of those who lost weight reported a reduction in appetite. Additionally, those who reported an increase in their desire for food (hunger) had twice the odds of weight gain (AOR = 2.19, 95% CI = 1.53, 3.14). Compared to those who didn't face any change, those who reported a decline in their desire for food (hunger) were more likely to lose weight (AOR = 0.54, 95% CI = 0.36, 0.81). These changes may be due to psychological and environmental stressors, consistent with other studies (46).

Regarding physical activity, the present study revealed that among the 1,682 participants, only 21.8% reported an increase in physical activity, 26.1% decreased their physical activity, 22.8% reported no changes, and 29.4% never practiced physical activity. Among those who gained weight, 36.5% reduced their levels of physical activity, and 30.5% had never practiced physical activity. These findings are consistent with recent studies highlighting many individuals (>50%) who reported changes in their physical activity and an increase in their sedentary behavior (8, 48, 49, 54). Previous studies have shown that reduced activity and increased sedentary time increase the risk of gaining weight both in general (55–57), and particularly during the COVID-19 pandemic, in both people with normal weight (4, 10, 58, 59) and with obesity (60).

Nevertheless, other studies have found that individuals had increased their physical activity levels during their lockdown periods. For example, 21.5% of those who maintained their weight had increased their level of physical activity, and 22.8% maintained the same level of physical activity. Among those who increased their physical activity (21.8%) during the lockdown, 38% lost weight, whereas 32% experienced no changes in their weight.

Physical activity was negatively associated with the change in weight. Increased physical activity increases the likelihood of weight loss (AOR = 0.61, 95% CI = 0.44, 0.86). On the other hand, those with lower physical activity levels had twice the odds of gaining weight (AOR = 2.25, 95% CI = 1.58, 3.21). This may be one of the ways of maintaining healthy behaviors and mitigating the negative impact of lockdown on mood and wellbeing (2, 55). Exercise positively impacts weight management, overall health, and mental wellbeing. It can improve mood, confidence, body image, motivation, and eating habits (61).

Results regarding smoking included that those who increased their smoking had a higher frequency of weight gain (5.0%) than those who decreased their smoking (3.1%). Results of logistic regression testing found the same association where those who smoked more were more likely to experience weight gain (AOR = 1.90, 95% CI = 1.04, 3.50). A similar study also showed a link between tobacco use and weight gain (62). Finally, the only finding regarding sleep was that those who sleep more than 9 h per night are more likely to lose weight (AOR = 1.90, 95% CI = 0.45, 0.88). Studies have found an association between sleep loss and irregular sleep, and weight gain during COVID-19 lockdowns around the world (63, 64).

Therefore, it is important to investigate the consequences of the COVID-19 pandemic and quarantine on the health of the UAE population to create targeted interventions to improve people's lifestyles following the pandemic and to prevent similar outcomes in the case of emergencies. This is especially needed in the UAE.

This study was subjected to several limitations. The study used snowball sampling by sending the survey to students who introduced an age bias. Most of the participants were women, as the survey was sent to Zayed University students who were mostly female. This makes the sample not representative of the UAE population. Another limitation is that the questionnaire questions were close-ended, meaning there might be other unexplored answers, and the choices might suggested answers that were otherwise not the participants' genuine opinions. Additionally, since it was an online questionnaire, the participants may have had a vague understanding of some questions, or they might have interpreted them differently than intended. However, this was minimized by using clear language and having the questions pilot tested by students that are not familiar with the subject (students not from the College of Natural and Health Sciences). Furthermore, this study did not examine the exact food habits and level of physical activity of the participants. Therefore, the data are lacking in these two areas, we didn't investigate all the components of Eating habits. For example, a Likert-type response of the consumption of specific food items, ranging from never to every day.

Overall, the present study found that most of the population has shown weight gain, increased food consumption, and decreased or no change in physical activity. The groups most likely to gain weight included those who consumed more meals and cereals, had an increased desire for sweets, and had an increased desire for food (hunger). In addition, changes in levels of physical activity were significantly associated with changes in weight during the lockdown. Those who exercised more were more likely to lose weight. Participants who slept more than 9 h were also more likely to lose weight. Finally, increases in smoking seem to be tied to weight gain. Interventions and awareness campaigns need to be conducted to encourage a healthier lifestyle for the people of the UAE. Not only is a healthy lifestyle important for immunity, it is also necessary to prevent chronic illnesses such as diabetes and heart disease. Preventing such illnesses has always been a goal of the UAE due to their high prevalence. Finally, further research can be done on the exact food habits and level of physical activity of the UAE population during the lockdown to find areas that need to be targeted.

## Data availability statement

The original contributions presented in the study are included in the article/supplementary material, further inquiries can be directed to the corresponding author.

## Ethics statement

This study was approved by the Research Ethics Committee at Zayed University, UAE (ZU20_137_F) and the Research Ethics Committee at the Ministry of Health and Prevention (MOHAP/DXBREC/ONN/No. 147/2020). All study participants provided informed consent at the beginning of the online questionnaire. The patients/participants provided their written informed consent to participate in this study.

## Author contributions

SA designed the study and manuscript writing. SA, MA, and AK recruited the participants and supervised the data collection. FE analyzed the data. SA, MA, ZT, and AK wrote the manuscript. All contributed authors of this original manuscript authorized the final version of the manuscript and read and approved the final version of the manuscript.
